# Lymphocytopenia and Anti-CD38 Directed Treatment Impact the Serological SARS-CoV-2 Response after Prime Boost Vaccination in Patients with Multiple Myeloma

**DOI:** 10.3390/jcm10235499

**Published:** 2021-11-24

**Authors:** Susanne Ghandili, Martin Schönlein, Christian Wiessner, Heiko Becher, Marc Lütgehetmann, Thomas Theo Brehm, Julian Schulze zur Wiesch, Carsten Bokemeyer, Marianne Sinn, Katja C. Weisel, Lisa B. Leypoldt

**Affiliations:** 1 The Department of Oncology, Hematology and Bone Marrow Transplantation with Section Pneumology, University Cancer Center Hamburg, 20251 Hamburg, Germany; m.schoenlein@uke.de (M.S.); c.bokemeyer@uke.de (C.B.); ma.sinn@uke.de (M.S.); k.weisel@uke.de (K.C.W.); l.leypoldt@uke.de (L.B.L.); 2 The Institute for Medical Biometry and Epidemiology, 20251 Hamburg, Germany; c.wiessner@uke.de (C.W.); h.becher@uke.dem (H.B.); 3 The Institute of Medical Microbiology, Virology and Hygiene, 20251 Hamburg, Germany; mluetgehetmann@uke.de; 4 The German Center for Infection Research (DZIF), Partner Site Hamburg-Lübeck-Borstel-Riems, 20251 Hamburg, Germany; t.brehm@uke.de (T.T.B.); j.schulze-zur-wiesch@uke.de (J.S.z.W.); 5 The I. Department of Internal Medicine, Division of Infectious Diseases, University Medical Center Hamburg-Eppendorf, Martinistraße 52, 20246 Hamburg, Germany

**Keywords:** multiple myeloma, SARS-CoV-2 vaccines, CD19+ B lymphocytes, anti-CD38-directed therapy, SARS-CoV-2 spike protein antibodies

## Abstract

Even though several SARS-CoV-2 vaccines have shown high effectiveness in the prevention of COVID-19 in healthy subjects, vaccination response in patients with plasma-cell-related disorders (PCD) remains widely unknown. Here, we report on an analysis describing the serological response after prime-boost SARS-CoV-2 vaccination in PCD patients, as compared to a healthy control group, and on possible influencing factors of serological responses. Blood samples were analyzed for the presence of quantitative anti-SARS-CoV-2 spike RBD Ig. A total of 82 patients were included; 67 received mRNA-, eight vector-based and four heterologous vaccinations. SARS-CoV-2 antibody titers (SP-AbT) were assessed in a mean of 23 days (SD ± 11 days) after the first and in a mean 21 days (SD ± 9) after prime-boost vaccination. A positive SP-AbT was detected in 31.9% of PCD patients after the first vaccination, and in 88.9% (44/49) after prime-boost vaccination, which was significantly less likely than that in the control group (100%, 78/78) (*p* = 0.008). Furthermore, we have been able to validate our previously suggested threshold of 30 CD19+ B lymphocytes/µL as being predictive for SP-AbT development. Despite anti-CD38 directed therapy, quadruplet treatment, higher age and missing deep remission, which correlated negatively with SP-AbT appearance, SP-AbT formation is possible in a majority of myeloma patients after prime-boost vaccination.

## 1. Introduction

The global impact of the current COVID-19 pandemic has led to an impressively rapid development and approval of multiple SARS-CoV-2 vaccines, which showed both a high efficacy in the prevention of COVID-19 and the reduction of disease severity in case of a breakthrough infection in healthy subjects [1,2,3,4]. Nevertheless, several trials have demonstrated significantly lower vaccine-induced seroconversion rates in patients with hematological malignancies when compared to solid cancer patients or healthy control groups [5,6]. Focusing on multiple myeloma (MM) patients, infections, in general, and COVID-19, in particular, pose a major threat due to plasma-cell-dysfunction-related immunodeficiency, based on suppression of CD19+ B lymphocytes, lower levels of polyclonal immunoglobulins, and both quantitative and functional T-cell abnormalities [7,8,9,10,11]. In line with this, a large retrospective analysis by the international myeloma society (IMS) revealed a considerably high COVID-19-related overall mortality rate of 33% in patients with underlying plasma cell disorders (PCDs) [12]. Previously published data investigating the efficacy of SARS-CoV-2 vaccines in patients with hematological malignancies indicate that the development of anti-SARS-CoV-2 antibodies is significantly impaired in myeloma patients when compared to healthy controls, with 15% of MM patients failing to develop a measurable serological immune response after prime-boost vaccination. Poor remission status, older age, type of anti-myeloma therapy and anti-CD38-directed therapy, in particular, have consistently been identified as additional factors impacting vaccination response [13,14,15,16,17,18,19]. However, since the expression of CD38 is not only limited to plasma cells but is also expressed on various immune cells, CD19+ B lymphocytes are targeted by anti-CD38-directed treatment as well [20,21,22]. Based on our previously published data, we were able to demonstrate for the first time that lower CD19+ B lymphocyte counts significantly correlate with poor anti-SARS-CoV-2-spike protein antibody titers (SP-AbT) after the first vaccination and identified a cutoff value of 30 CD19+ cells/µL to predict a serological response. Furthermore, we were able to highlight the negative correlation of anti-CD38-directed treatment with poor SP-AbT after receiving the first SARS-CoV-2 vaccine in patients with underlying PCD [23].

Here, we discuss a detailed analysis regarding the development of SP-AbT after prime-boost vaccination in patients with PCD and a comparison with healthy controls.

## 2. Materials and Methods

### 2.1. Study Design and Patients

This prospective observational study included patients who met all of the following criteria:

(a) Aged 18 years and older;
(b) Had a confirmed diagnosis of MM, smoldering MM (sMM), monoclonal gammopathy of clinical significance (MGCS) and systematic light chain amyloidosis (AL) according to the 2014 updated diagnostic criteria of the International Myeloma Working Group (IMWG) [24];
(c) Were eligible for anti-SARS-CoV-2 vaccination according to IMS recommendations [24,25];
(d) Provided written informed consent.

Furthermore, healthcare workers of the same sex and vaccination regime were selected as a healthy comparison group and included in the current analysis [26]. This single-center analysis was performed between January 1st and July 30th 2021 at the department of oncology and hematology of the University Medical Center Hamburg-Eppendorf, Germany, and included partially the same patient cohort as in our previously reported analysis [23]. Exclusion criteria were a prior confirmed infection with SARS-CoV-2, defined as either a polymerase-chain-reaction-test-validated infection or evidence of anti-SARS-CoV-2 nucleocapsid and spike protein antibodies before immunizations.

The primary aim of this study was to evaluate a possible correlation between SP-AbT after prime-boost vaccination and CD19+ B lymphocyte count in patients with underlying PCD. Moreover, we aimed to compare the number of subjects who developed SP-AbT after prime-boost vaccination, as well as the level of SP-AbT after prime-boost vaccination between patients with PCD and a healthy control group. SARS-CoV-2 vaccines used in this trial were Comirnaty^®^ (previously: BNT162b2 by BioNTech, Mainz, Germany; Pfizer, New York City, NY, USA), and Moderna vaccine (previously: mRNA-1273 by Moderna, Cambridge, MA, USA), summarized as mRNA-vaccines, and Vaxzevria^®^ (previously: AZD1222 by AstraZeneca, Oxford, United Kingdome) referred to as vector-based vaccines. No data were collected regarding vaccination side effects or toxicities.

Myeloma-directed treatment was adapted according to the recommendations of the IMS in routine clinical care [24]. Clinical data regarding treatment and disease characterization were collected from the patient’s electronic medical records. For statistical analysis, groups of deep remission status (≥very good partial response (VGPR)) and without deep controlled disease (≤partial response) at the time of vaccination were defined.

This study is part of the COVIDOUT trial registered at ClinicalTrials.gov (NCT04779346) and was approved by the Ethics Committee of the Medical Council of Hamburg (reference 2020-10275-BO-ff). Written informed consent was provided by each patient.

### 2.2. Detection of Anti-SARS-CoV-2 Antibodies

Blood samples were analyzed for the presence of SARS-CoV-2 specific antibodies, using the quantitative anti-spike Ig (SARS-CoV-2 spike RBD Ig, cutoff ≥ 0.8 AU/mL) and qualitative anti-NC Ig assay (Elecsys Anti-SARS-CoV-2, Roche; cutoff ≥ 1 COI). A mathematical transposition of Elecsys SARS-CoV-2 spike RBD Ig specific AU/mL to the World Health Organization standard BAU/mL was performed by using the following equation: AU/mL = 0.972 × BAU/mL [27].

Clinical laboratory improvement amendments were performed by using the immuno-analyzer (Cobas e411, Roche, Mannheim, Germany; and Liaison XL, DiaSorin, Salluggia, Italy) according to the manufacturer’s recommendations.

### 2.3. Flow Cytometry Procedure

Flow cytometric analyses were performed for assessment of the patients’ lymphocyte status on Navios flow cytometer with CXP Software Version 2.0(Beckmann Coulter Krefeld, Germany), as previously described [23].

### 2.4. Statistical Analysis

In the myeloma patient cohort, the relationship between SP-AbT and variables of interest (CD19+ B cell count, age, vaccination type, time since vaccination, quadruplet therapy and deep remission) was evaluated by bivariate analysis and multivariable linear regression. The antibody levels of myeloma patients and healthy controls were compared by multivariable linear regression, adjusting for demographic variables, vaccination type and time since vaccination. Bivariate comparisons were performed by using the Mann–Whitney U test for continuous characteristics, and by Fisher’s exact test for categorical characteristics, while multivariate associations were analyzed by linear regression. Due to the highly skewed distribution of CD19+ cell count and SP-AbT, both variables were log-transformed for analysis. A *p*-value < 0.05 was considered statistically significant. The reported *p*-values are two-tailed. All statistical analyses were performed, and figures were designed by using the Statistical Package for Social Sciences statistical software, version 26.0 (IBM Corp., Armonk, NY, USA) and by SAS software (version 9.4 of the SAS System for Windows; SAS Institute, Inc., Cary, NC, USA) and GraphPad Prism, version 9 for macOS (GraphPad Software, La Jolla, CA, USA).

## 3. Results

A total of 82 patients was included in this observational trial. Of those, 67 patients received mRNA-, eight vector-based and four heterologous vaccinations. The median interval between the first and second vaccination were 40 days (r: 19–49) in patients receiving mRNA-, 84 days (r: 71–85) receiving vector-based and 84 days (r: 76–84) in those receiving heterologous vaccination, respectively. There are no data on the vaccination schedule available for three patients. Patient demographics and characteristics are presented in Table 1. The median age was 68 years (range (r): 35–85). Seventy-four patients had MM, four sMM or MGCS, and four AL (overall summarized as PCD). In total, 37 patients (45.1%) received anti-CD38-targeting and two (2.4%) received anti-SLAMF7-targeting therapies at the time of vaccination, 52 (63.4%) patients received immunomodulatory drug (IMiD)-based treatments and 13 patients (15.9%) were under active surveillance without current treatment. Thirteen patients (15.9%) received quadruplet treatment consisting of a combination of daratumumab, bortezomib, thalidomide and dexamethasone (DaraVTd); isatuximab, carfilzomib, lenalidomide and dexamethasone (IsaKRd); or elotuzumab, pomalidomide, cyclophosphamide and dexamethasone (Elo-PCd). A total of 52.4% of patients had newly diagnosed and 41.5% had refractory or relapsed (R/R) disease. Overall, 75.6% of all patients were in deep remission (≥VGPR) at the time of both vaccinations.

The assessment of anti-SARS-CoV-2 antibody titers in myeloma patients took place, on average, 24 days (standard deviation (SD) ± 11.2 days, range: 8–63 days) after the first and 21 days (SD ± 9.4 days, range: 6–53) after the prime-boost vaccination. A positive SP-AbT was detected in 31.9% of assessable patients, with an overall median SP-AbT of 0 BAU/mL (r: 0–10328, mean 202.36) after the first vaccination and increased up to 88.9% (median SP-AbT of 216.87 BAU/mL; r = 0–25720; mean, 2139.29) after prime-boost vaccination. Of the myeloma patients not showing positive SP-AbT after the first vaccination, 80.9% became positive after prime-boost vaccination, while 19.1% remained negative. For those patients who became positive only after the prime-boost vaccination, median SP-AbT titer was significantly lower compared to those patients who became positive after the first vaccination (51.04 vs. 2191.87 BAU/mL, *p* < 0.0001).

Immunophenotypic analysis was performed for differentiation of T- and B-cell subsets. Here, a CD19+ B-cell count of median 33.5/µL (r: 1–696/µL) was seen in the overall myeloma patient cohort; in patients with negative SARS-CoV-2 SP-AbT, the median CD19+ B cell numbers were significantly lower compared to patients with positive titers (median CD19+ B cells: 2.0 vs. 52.5/µL, *p* = 0.005). Overall, CD19+ B lymphocyte numbers significantly correlated with positive SP-AbT results and were identified as a predictive factor in multivariate analysis. The previously suggested threshold of 30 CD19+ B cells/µL as being predictive for SP-AbT development could be validated [23]. However, CD4+ and CD8+ T-cell numbers did not correlate with the SP-AbT concentration.

**Figure 1:**
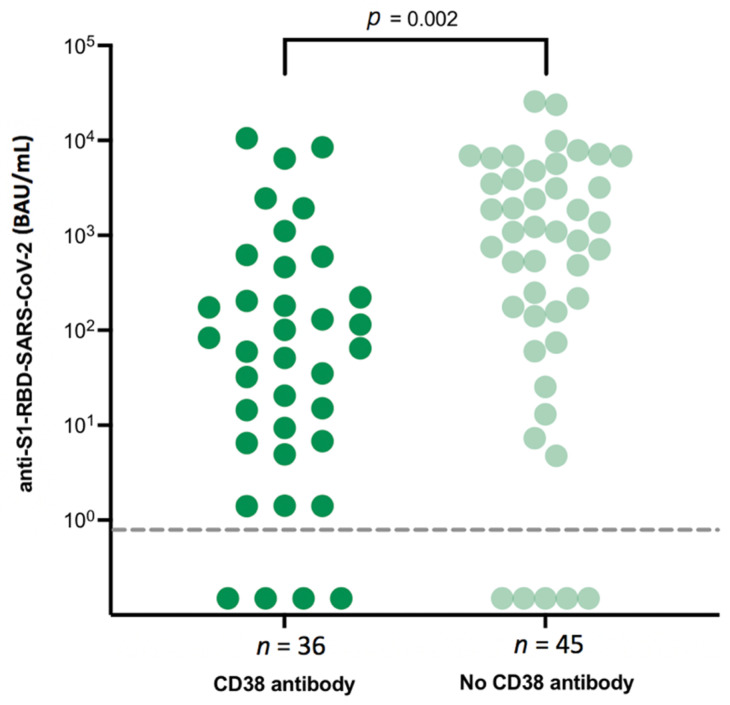
Serological response depending on concomitant anti-CD38-directed treatment.

Next, we investigated the impact of different anti-myeloma treatments on the level of SP-AbT after prime-boost vaccination. An IMiD-based therapy did not influence the level of SP-AbT compared to patients who did not receive an IMiD-based therapy (median SP-AbT for IMiD-based therapy vs. non-IMiD-based therapy: 157 vs. 608 BAU/mL; *p* = 0.1). However, anti-CD38-directed treatment did negatively impact the serological response: patients under anti-CD38-directed treatment showed significantly lower median SP-AbT when compared to those without (median SP-AbT for anti-CD38-directed treatment: 62 vs. 1085 BAU/mL, *p* = 0.002) (Figure 1).

**Figure 2:**
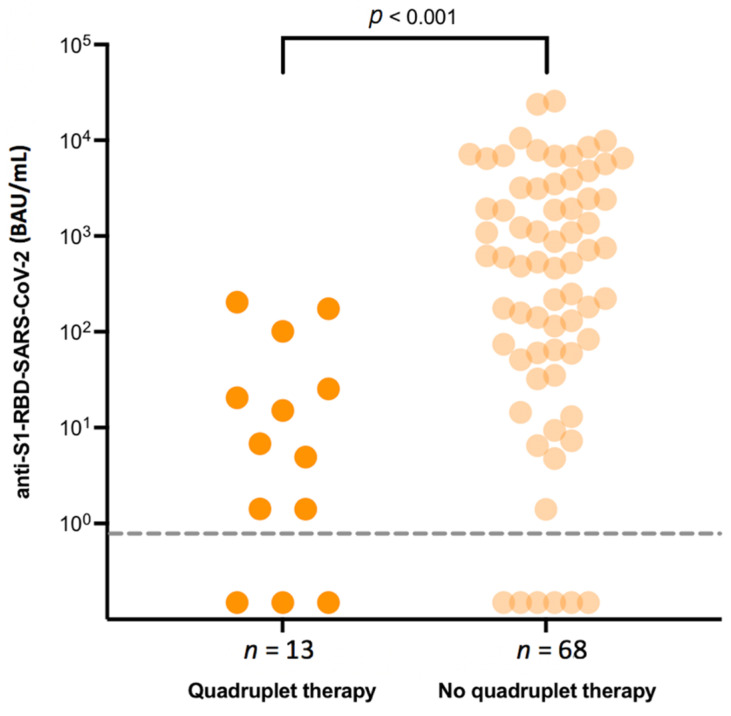
Serological response depending on concomitant quadruplet treatment.

Any quadruplet therapies consisting of either DaraVTd and IsaKRd as first-line treatment for patients with newly diagnosed MM or Elo-PCd in R/R disease did significantly negatively impact the development of SP-AbT (median SP-AbT for quadruplet therapy vs. non-quadruplet therapy: 7 vs. 565 BAU/mL, *p* < 0.001) (Figure 2).

Moreover, patients with deep responses developed significantly higher levels of SP-AbT when compared to patients with partial response (PR), stable disease or progressive disease (PD), respectively (median SP-AbT for deep remission vs. PR, stable disease, or PD: 248 vs. 13 BAU/mL, *p* = 0.023). The patients’ sex did not significantly impact the level of SP-AbT (median SARS-CoV-2 SP-AbT for male vs. female: 509 vs. 130 BAU/mL, *p* = 0.1), while SP-AbT concentration was significantly lower with older age.

For further evaluation of underlying factors possibly impacting vaccination response, we performed a multivariate analysis. In our multiple linear regression analysis, the SP-AbT concentration was significantly lower in patients with increasing age (coefficient −0.10, 95% confidence interval [−0.14; −0.06] *p* < 0.001), and under quadruplet treatment (−1.44; [−2.75;−0.13] *p* = 0.032) (Figure 2). In contrast, logarithmized CD19+ B-lymphocyte count was positively correlated with SP-AbT (1.01; [0.71; 1.30] *p* < 0.001) (Table 2).

Due to the small number of patients not developing SP-AbT after the second vaccination, statistical analysis regarding impacting factors to characterize poor responders in advance was statistically not reliable because of large confidence intervals. A purely descriptive analysis of the non-responders is given in Table 3. All non-responders showed at least two of the five previously described characteristics of a poor vaccination response: (a) age ≥ 65 years, (b) ≤ 30 CD19+ B lymphocytes/µL, (c) current anti-CD38-directed treatment, (d) any quadruplet treatment and (e) not in deep remission (≤PR). In a total of three patients, the vaccination schedule remains unknown. However, all three patients developed a positive SP-AbT after their prime-boost vaccination.

In order to be able to make a reliable comparison between the group of myeloma patients and a healthy control group (consisting of healthcare workers), both groups were adjusted with regard to the frequency of vaccination regime. Due to the maximum age of 69 years in the control group and the described negative effect of older age on SARS-CoV-2 SP-AbTs, myeloma patients older than 70 years were not included in this comparative analysis. Thus, a total of 49 myeloma patients and 78 healthy controls were included. Demographics and characteristics of both groups are presented in Table 4.

**Figure 3:**
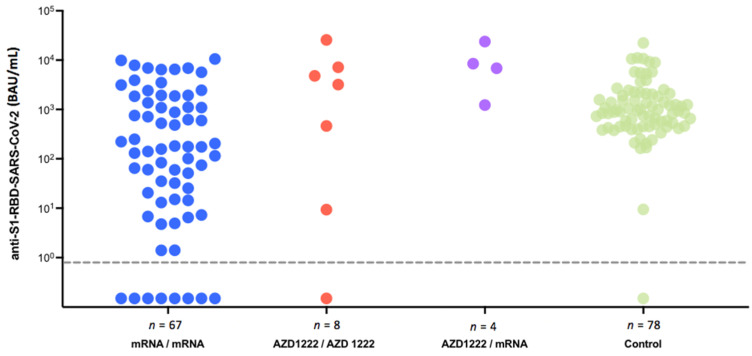
Serological response depending on vaccination type.

A positive SP-AbT after prime-boost vaccination was significantly less likely to be detectable in myeloma patients with 44 of the 49 patients (89.9%) than in the control group with 78 of the 78 controls (100%) (*p* = 0.008) (Figure 3). There were no significant differences regarding the overall median SP-AbT (Myeloma patients: 878 BAU/mL (r: 0–25,720); healthy controls: 938 BAU/mL (r: 0.3–22,287), *p* = 0.64). Moreover, in the multivariate analysis, this younger subgroup of myeloma patients did not differ in their antibody titers compared to the control group (β = −0.65, 95% confidence interval [−1.99; 0.69]). The only significant predictor of the antibody concentration was the vaccine type since study participants receiving a heterologous vaccination regimen had significantly higher antibody concentrations.

## 4. Discussion

Even though several SARS-CoV-2 vaccines have shown high effectiveness in the prevention of COVID-19 in healthy subjects, the vaccination response and its mediated protection against SARS-CoV-2 infection in patients with MM and other PCD remains widely unknown. We have compared the proportion of myeloma patients with a positive SP-AbT after the first and second vaccinations. The comparison has shown an increase in serological vaccination response from 31.9% after the first vaccination to 88.9% after the prime-boost vaccination (with positive SARS-CoV-2 SP-AbT). However, compared to healthy controls with vaccination response rates of ≥90%, this leaves about 5–22% of myeloma patients without protection against COVID-19 despite prime-boost vaccination [14,15,17,28,29]. Our results are in line with those of Pimpinelli et al., who observed a significant increase of serological response from 21.4% after the first dose of mRNA vaccine to 78.6% after the second dose of mRNA vaccine in 42 patients with MM, also in line with Van Oekelen et al., according to whom, 84.2% (219/260) of myeloma patients developed detectable SP-AbT at least 10 days after prime-boost vaccination [15,17]. Recently, Greenberger et al. investigated the serological vaccination response in 1495 patients with hematological malignancies, including 184 patients with MM 14 days after receiving the second dose of a mRNA vaccine. Seronegativity was described in 5.3% of patients with MM [29]. However, since there are no detailed data provided by Greenberger et al. regarding the kind of anti-myeloma treatment and remission status, we assume that the differences in terms of seronegativity rates could be based on smaller proportions of myeloma patients under active treatment, particularly anti-CD38-directed treatment, and possibly more myeloma patients in deep remission, as every single one of these factors is known to impact vaccination response.

We have also demonstrated the negative impact of an anti-CD-38-directed therapy on vaccination response by observing significantly lower median SP-AbT in patients who are currently receiving anti-CD-38 directed treatment when compared to those without, which is in line with previously published data [15,16,17]. Furthermore, for the very first time, we have been able to carve out a significantly negative impact of quadruplet therapies on the development of SARS-CoV-2 SP-AbT. In addition, in line with recommendations of the IMS, concurrent treatment with IMiDs did not impair vaccination response and may thus be continued throughout the vaccination process.

The presence of lymphocytopenia at the time of vaccination as a significant predictive factor for a decreased humoral response after prime-boost vaccination-related immunization against SARS-CoV-2 has been previously reported [14,16,17]. By using the more precise differentiation of lymphocytes by flow cytometry analysis, we have been able to demonstrate that a count of at least 30 CD19+ B-lymphocytes/µL correlates significantly with a positive SP-AbT and identified the previously suggested threshold of 30 CD19+ B lymphocytes/µL as being predictive for SP-AbT development in patients with MM and other PCD [23]. CD19+ B lymphocyte count may be of use as another parameter to assess the probability of a strong immune response or to identify patients with expected impaired response.

While vaccination response rates are significantly lower in MM patients, median SP-AbT levels of the younger patient subgroup and healthy controls were not significantly different. Based on these data, it remains unanswered whether this observation is also found in older myeloma patients. With growing data underlining that higher SP-AbT levels are more protective than lower titer levels and with the heterologous vaccination leading to significantly higher SP-AbT levels, a new debate about recommending heterologous vaccination to immunocompromised patients may be opened [30].

Albeit the size of the current MM patient cohort was too small to statistically evaluate a predictive score for the likelihood of vaccination response, we suggest to be particularly alert in patients in whom two or more of the following factors apply: (a) age ≥ 65 years, (b) ≤ 30 CD19+ B lymphocytes/µL, (c) current anti-CD38-directed treatment, (d) any quadruplet treatment and (e) not in deep remission (≤PR). For those patients, intensified vaccination regimes, possibly with heterologous vaccination, may be of high importance to induce sufficient SARS-CoV-2 SP-AbT. The recently granted approval of booster immunizations by the US Food and Drug Administration for immunocompromised patients seems thus sensible and does hopefully help to booster vaccination responses [31].

Although this was a prospectively aimed observational trial, there are some limitations. Based on the comparatively small cohort size, the characterization of vaccination non-responders could only be performed descriptively. In addition, since assessments were performed as part of clinical routine in a real-world setting, the time points of SP-AbT measurement after vaccinations were not standardized and took place within a wider range. In some MM patients, the response assessment took place rather early after vaccination, especially in one patient with assessment on day 6, which may have impacted SP-AbT formation. In contrast, measurement of SP-AbT in the control group was performed on later timepoints. However, with the data showing constant SP-AbT for several months after vaccination, we deem the control group valid for comparison [32]. In addition, our analyses did not include T-cell functioning tests or an assessment of neutralizing SARS-CoV-2 antibody titer, which are both known to be of importance for mediating immunity [33].

Overall, this analysis of SP-AbT in patients with PCD together with the assessment of CD19+ B-lymphocyte counts underlines the importance of at least 30 CD19+ cells/µL for sufficient vaccination response and helps to further characterize factors impacting SP-AbT formation in MM patients with the most important factors being older age, anti-CD38-directed treatment, poor remission status and quadruplet treatment as newly identified factors. While COVID-19 will remain a relevant risk for patients with PCD, this and other studies have by now helped to provide more detailed information on who will most likely not be sufficiently protected by vaccinations and thus needs mitigation with intensified vaccination strategies, the creation of herd immunity and ring vaccination of the patients’ environment, possible prophylactic use of monoclonal antibodies against SARS-CoV-2 spike protein and maintaining of protective measures. However, while only about 20–30% of myeloma patients developed positive SP-AbT after the first vaccination, this increased to a promising 88% after prime-boost vaccination, indicating that sufficient SP-AbT formation is possible also in MM patients and may be further enhanced by upcoming booster vaccinations.

## Figures and Tables

**Table 1:** Myeloma patients’ demographics and characteristics.

Variable, *n* (%) If Not Other Identified	Total
Age, median age in years (range)	68 (35–85)
Male sex	49 (60)
**Type of plasma-cell-related** **neoplasia**	
MM	74 (90.2)
sMM	2 (2.4)
MGCS	2 (2.4)
AL	4 (4.9)
Newly diagnosed	48 (58.5)
Refractory or relapsed	34 (41.4)
Therapy lines, median number in total (range)	1 (0–8)
**Anti-myeloma therapy**	
Anti-CD38 directed therapy	37 (45.1)
Daratumumab-based	27
Isatuximab-based	10
Elotuzumab-based	2 (2.4)
IMiD-based therapies in total	52 (63.4)
Thalidomide-based	1
Lenalidomide-based	48
Pomalidomide-based	4
Proteasome inhibitor-based in total	17 (20.7)
Bortezomib-based	4
Carfilzomib-based	13
No current therapy	13 (15.9)
Quadruplet treatment	13 (15.9)
Remission status: deep remission ≥ VGPR	62 (75.6)
**Vaccination type**	
mRNA-based	67
Vector-based	8
Heterologous	4

**Table 2:** Multiple linear regression of factors associated with logarithmized antibody titers in myeloma patients.

Parameter	Coefficient	95% Confidence Interval	*p*-Value
Constant	6.83	[3.54; 10.10]	<0.001
CD 19 + B cells (logarithmized)	1.01	[0.71; 1.30]	<0.001
Age	−0.10	[−0.14; −0.06]	<0.001
Vaccine (reference: mRNA) Vector Heterologous	0.30 2.73	[−1.18; 1.78] [0.11; 5.36]	0.69 0.042
Days post second vaccination	0.03	[−0.02; 0.08]	0.19
Quadruplet therapy (reference: other therapy)	−1.44	[−2.75; −0.13]	0.032
Deep remission ≥ VGPR (reference: ≤ PR)	0.87	[−0.37; 2.12]	0.17

**Table 3:** Descriptive characterization of non-responders.

	Age (Years)	Sex	CD19+ Cells/µL	Vaccine Type	Disease Type	ND/RR	Current Treatment	Line of Therapy	Remission
I	69	female	2	mRNA	MM	RR	Elo-PCd	5	SD
II	61	male	1	vector	MM	ND	IsaKRd	1	sCR
III	68	female	9	mRNA	MM	RR	Rd	2	VGPR
IV	78	female	11	mRNA	MM	RR	PCd	8	PD
V	66	female	1	mRNA	MM/AL	RR	DRd	2	VGPR
VI	81	male	9	mRNA	MM	ND	DRd	1	VGPR
VII	63	female	1	mRNA	MM	ND	IsaKRd	1	sCR
VIII	78	male	1	mRNA	MM	RR	PCd	3	PR
IX	76	male	19	mRNA	MGCS (renal)	ND	Vd	1	n/a

MM, multiple myeloma; MGCS, mono-clonal gammopathy of clinical significance; DRd, daratumumab, lenalidomide and dexame-thasone; Elo-PCd, elotuzumab, pomalidomide, cyclophosphamide and dexamethasone; IsaKRd, isatuximab, carfilzomib, lenalidomide and dexamethasone; PCd, pomalidomide, cyclophospha-mide and dexamethasone; Rd, lenalidomide and dexamethasone; Vd, bortezomib and dexame-thasone.

**Table 4:** Demographics and characteristics of myeloma-patient subgroup and control group (≤70 years); SD, standard deviation.

Variable, *n* (%) If Not Other Identified	Myeloma-Patient Group (*n* = 49)	Control *Group* (*n* = 78)	*p*-Value
Mean age in years (SD)	59.6 (8.4)	51.3 (7.5)	<0.001
Male sex	28 (57%)	45 (58%)	0.95
Days post second vaccination, mean (SD)	20.4 (9.0)	88.0 (37.2)	<0.001
Vaccination type			0.78
mRNA-based	39 (81%)	67 (86%)	
Vector-based	6 (13%)	7 (9%)	
Heterologous	3 (6%)	4 (5%)	
TOTAL	*n* = 49	*n* = 78	
